# Chronic Dialysis Dependent Renal Failure Resulting from a Massive Bladder Containing Inguinal Hernia

**DOI:** 10.1155/2017/2368237

**Published:** 2017-05-07

**Authors:** Michael M. Herskowitz, Jamel Reid, Robert F. Leonardo

**Affiliations:** Department of Radiology, SUNY, HSBC, 450 Clarkson Avenue, Brooklyn, NY 11215, USA

## Abstract

Bladder involvement in inguinal hernia is relatively rare, 1–4%, although the incidence is increased to 10% with advancing age or obesity. There are several previously reported cases presenting with obstructive uropathy and renal failure, but all reversed with urinary diversion and hernia repair. We believe this to be the first reported case of bladder hernia leading to dialysis dependent chronic renal failure.

## 1. Introduction

Inguinal hernias are the most common form of abdominal wall hernias. Typical contents include abdominal fat and bowel. Inguinal hernias with bladder involvement are relatively rare entities. Several cases have been previously reported with obstructive uropathy. We present a rare case of bladder containing inguinal hernia resulting in chronic dialysis dependent renal failure, which we believe is the first reported case of its kind.

## 2. Case Presentation

A 59-year-old Haitian-American male presented to the emergency room in hypertensive crisis, complaining of dizziness, with a blood pressure of 240/120. He also related lower urinary tract symptoms to difficulty in initiating urination and incomplete bladder emptying. He stated that there has been slow gradual enlargement of a right inguinal mass over the past year. Physical examination revealed the large right inguinal hernia. Laboratory evaluation was remarkable for BUN/Creat. of 51/7.1 and Hgb/Hct of 10.2/31.2.

**Figure 1:**
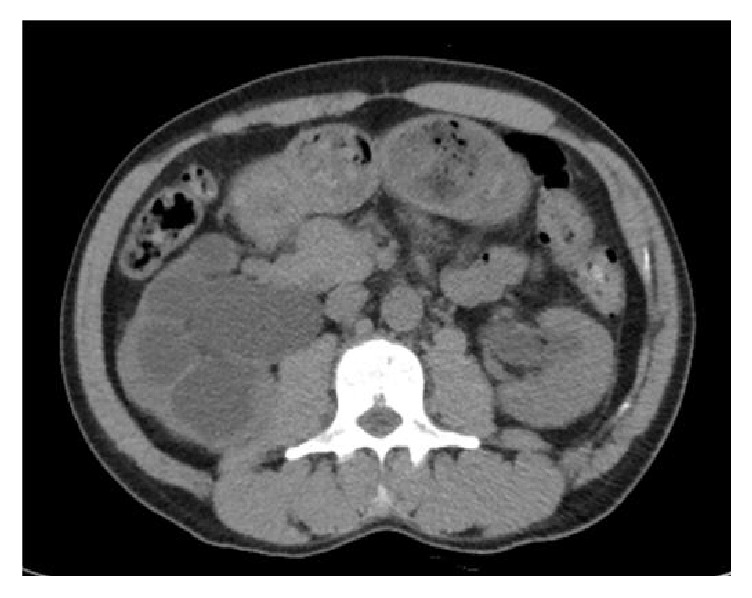
CT scan at the time of admission taken at the level of the kidneys reveals bilateral hydronephrosis, the right greater than the left.

**Figure 2:**
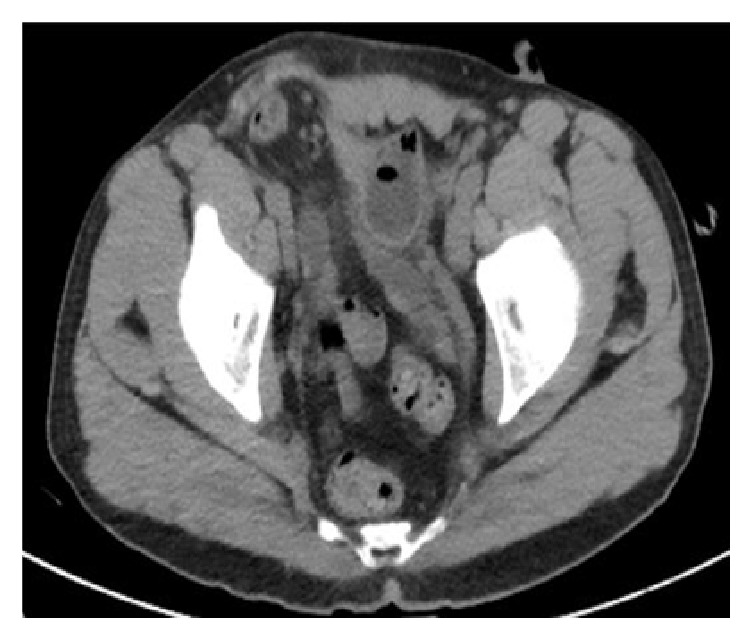
CT image demonstrates narrowing of bilateral dilated ureters entering the inguinal canal.

**Figure 3:**
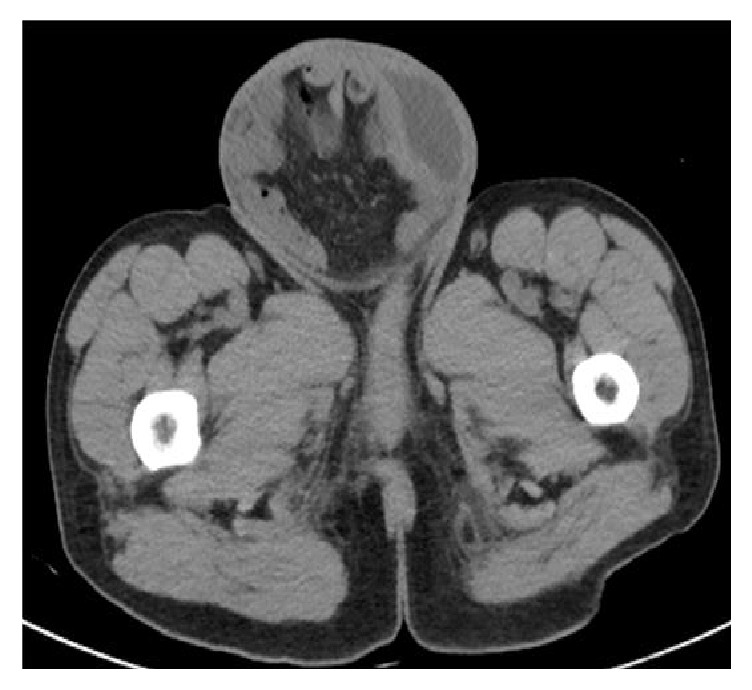
A more inferior CT image demonstrates a portion of the bladder within the very large right inguinal hernia.

A CT scan of the abdomen and pelvis showed bilateral hydronephrosis, the right greater than the left, a large right inguinal containing bladder, and narrowing of bilateral dilated ureters entering the inguinal canal (Figures 1–3).

The patient underwent bilateral percutaneous nephrostomy placement the following day. The creatinine level remained essentially unchanged. On hospital day # 9, the patient underwent right inguinal hernia repair with mesh and replacement of the bladder into the pelvis. On hospital day # 18 the BUN/Creat. remained 56/6.3 and the creatinine never fell below 5.7. On hospital day # 21, bilateral nephrostograms were performed which showed patent ureters with filling of the bladder, now restored to its normal position within the midline pelvis. Both nephrostomy tubes were removed. On hospital day # 31, with no significant improvement in renal function, a tunneled jugular dialysis catheter was placed with plans for fistula creation, and dialysis was initiated.

## 3. Discussion

Bladder involvement in inguinal hernia is relatively rare (1–4%) [1–3], although the incidence in the elderly may approach 10%. Risk factors also include obesity and weakening of the lower abdominal musculature and bladder outlet obstruction. There is also a higher incidence of genitourinary malignancies in these patients [2]. The bladder related hernias are more often direct than indirect, inguinal more than femoral, and more often right-sided and have a male predominance of 70%. The bladder involvement in the majority of these is not diagnosed before surgery, only becoming obvious during the operative period [3]. Clinical abnormalities associated with bladder hernias include unilateral or bilateral hydronephrosis, bladder stones, ureteral reflux, and renal failure.

Surgical repair is recommended for all bladder hernias, with preoperative percutaneous nephrostomy indicated for cases with significant hydronephrosis. The entire bladder should be replaced into the pelvis with partial bladder resection recommended only in cases of bladder neoplasm, necrosis, or bladder diverticulum involved in the hernia [5].

Several authors have reported cases of patients with bladder hernias presenting with obstructive uropathy and renal failure [4–6]. However, all of these patients returned to normal renal function after completion of all interventions. We believe this to be the first reported such case leading to chronic dialysis dependent renal failure. This underscores the need for patients to seek investigation of and treatment of large inguinal hernias, especially those involving the bladder, to prevent long term complications.

## 4. Conclusion

We present a rare case of massive inguinal scrotal hernia containing bladder. This patient presented with obstructive uropathy and renal failure, which did not reverse with appropriate therapies. We believe this is the first reported case of chronic dialysis dependent renal failure associated with inguinoscrotal bladder hernia.
